# Homing of stem cells to sites of inflammatory brain injury after intracerebral and intravenous administration: a longitudinal imaging study

**DOI:** 10.1186/scrt17

**Published:** 2010-06-15

**Authors:** Johanna S Jackson, Jon P Golding, Catherine Chapon, William A Jones, Kishore K Bhakoo

**Affiliations:** 1 Stem Cell Imaging, MRC Clinical Sciences Centre, Imperial College London, Du Cane Road, London W12 0NN, UK; 2 Department of Life Sciences, The Open University, Walton Hall, Milton Keynes MK7 6AA, UK; 3 Singapore Bioimaging Consortium (SBIC), Agency for Science, Technology and Research (A*STAR), 11 Biopolis Way, 02-02 Helios, 138667 Singapore

## Abstract

**Introduction:**

This study aimed to determine the homing potential and fate of epidermal neural crest stem cells (eNCSCs) derived from hair follicles, and bone marrow-derived stem cells (BMSCs) of mesenchymal origin, in a lipopolysaccharide (LPS)-induced inflammatory lesion model in the rat brain. Both eNCSCs and BMSCs are easily accessible from adult tissues by using minimally invasive procedures and can differentiate into a variety of neuroglial lineages. Thus, these cells have the potential to be used in autologous cell-replacement therapies, minimizing immune rejection, and engineered to secrete a variety of molecules.

**Methods:**

Both eNCSCs and BMSCs were prelabeled with iron-oxide nanoparticles (IO-TAT-FITC) and implanted either onto the corpus callosum in healthy or LPS-lesioned animals or intravenously into lesioned animals. Both cell types were tracked longitudinally *in vivo *by using magnetic resonance imaging (MRI) for up to 30 days and confirmed by postmortem immunohistochemistry.

**Results:**

Transplanted cells in nonlesioned animals remained localized along the corpus callosum. Cells implanted distally from an LPS lesion (either intracerebrally or intravenously) migrated only toward the lesion, as seen by the localized MRI signal void. Fluorescence microscopy of the FITC tag on the nanoparticles confirmed the *in vivo *MRI data,

**Conclusions:**

This study demonstrated that both cell types can be tracked *in vivo *by using noninvasive MRI and have pathotropic properties toward an inflammatory lesion in the brain. As these cells differentiate into the glial phenotype and are derived from adult tissues, they offer a viable alternative autologous stem cell source and gene-targeting potential for neurodegenerative and demyelinating pathologies.

## Introduction

A number of CNS injury and neurodegenerative diseases result in varying degrees of cell death and neuroinflammation. Cell-based therapies involving stem cells aim to replace these lost cells or repair damaged areas, thus providing functional recovery. However, before the potential of stem cell-based therapies can be realized, it is important to understand the behavior of these cells after implantation *in vivo *and the practicalities of different routes of administration. Such longitudinal studies are best performed by using noninvasive *in vivo *MRI cell-tracking protocols and indeed are a prerequisite for cell-replacement therapy for translation to the clinic.

An important factor in clinical translation of cell-based therapy is the selection of appropriate cell types for transplantation. Stem cells derived from adult tissues have a number of advantages, including their utility in autologous stem cell therapy, where they not only reduce the need for immunosuppression but are harvested relatively easily in the clinic. Current literature indicates the promising repair potential of two adult stem cell types: epidermal neural crest cells and bone marrow mesenchymal stem cells. Neural crest cells (NCCs) constitute a population of neuroepithelial, migratory, multipotent stem cells that give rise to most of the peripheral nervous system during development, including dorsal root ganglia (DRG) neurons, DRG satellite glia, and Schwann cells. NCCs also give rise to melanocytes and skeletal structures of the face. Until recently, NCCs were considered a transient cell population that existed only during development. However, recent work demonstrated that multipotent NCCs persist into adulthood within the stem-cell niche of hair follicles, and these adult-resident cells have been termed epidermal neural crest stem cells (eNCSC) [1]. Because eNCSCs retain the neuroglial differentiation potential of embryonic NCCs [1] and can be harvested from hairy skin by using a minimally invasive procedure, they are an attractive cell type to consider for autologous cell-replacement therapies to repair the injured CNS.

The other cell type with a similar potential for autologous cell-based applications in CNS repair is mesenchymal stem cells (MSCs) derived from the bone marrow. MSCs derived from marrow stromal cells differentiate into tissues of mesodermal lineage, such as fat, bone, and cartilage [2], but can also differentiate along neuroglial lineages [3]. In the uninjured rat brain, bone marrow-resident cells continuously contribute to neural populations, including neurons, microglia, and occasionally astrocytes in the dentate gyrus and periventricular zones [4]. However, the numbers of resident MSCs homing to CNS lesions are small. By harvesting, expanding *in vitro*, and then re-introducing autologous cells systemically, more stem cells are likely to home to the sites of CNS injuries, where they can provide therapeutic benefit. For example, human MSCs injected intravenously into rats after spinal cord injury were found to infiltrate mainly into the ventrolateral white matter tracts, where they differentiated into oligodendrocytes [5]; however, the migration of the cells was assessed only at the end point instead of the longitudinal imaging used in the present study. In stroke and inflammatory-lesion models of adult brain injury in rats, as well as in human stroke studies, injected MSCs have been reported enter the brain from the circulation and stimulate host oligodendrogenesis and remyelination [6-9]. Although it must be emphasized that the differentiation of BMSCs into neural phenotypes is somewhat controversial, their homing ability may be useful in gene or growth-factor delivery.

Several studies have demonstrated the migration of stem cells after intravenous injection (Chen *et al.*, 2001; Garbuzova-Davis *et al.*, 2003; Jendelova *et al.*, 2004; Takeuchi *et al.*, 2007) in models of neurodegeneration. However, the models used in these studies involved mainly ischemia and compression injury, which give rise to more severe lesions. This study aims to assess stem cell behavior in more moderate cerebral insults. In addition, although earlier studies clearly demonstrate the potential of eNCSCs and BMSCs for neural repair, they provide only a 'snapshot,' postmortem view of what is happening to the transplanted cells. Therefore, the aims of this study were to use longitudinal *in vivo *MR imaging, followed by histologic analysis, to monitor the migratory potential and resulting fate of eNCSCs and BMSCs, by using two routes of administration (cerebral implantation or intravenous injection) in a lipopolysaccharide (LPS) lesion model of CNS inflammation.

## Materials and methods

### Animals

All surgical procedures were carried out under a UK Home Office Project Licence in accordance with the UK (Animals) Scientific Procedures Act 1986. Healthy adult (250 to 300 g) male Sprague-Dawley rats (Harlan, UK) were acclimatized for a week on a regular diet and equal light/dark cycle.

### Cell purification

**Figure 1:**
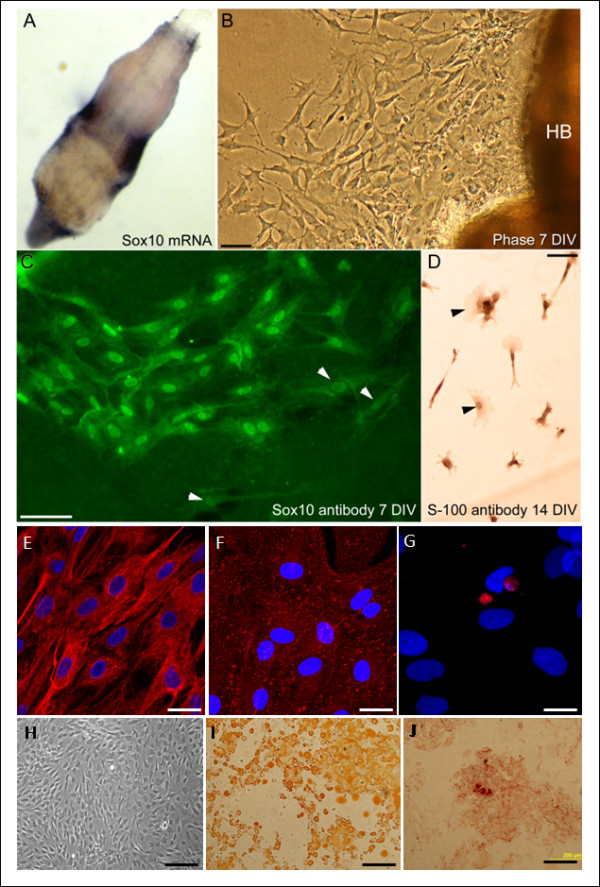
**Phenotype of implanted cells**. **(a-d) **Neural crest stem cells (NCSCs); **(e-j) **bone marrow stem cells (BMSCs). (a) Sox10 *in situ *of the bulge region of a hair bulb, revealing the NCSC population. (b) Cell emigration from a hair bulb (HB) after 7 days *in vitro*. (c) Sox10 immunostaining of the NCSCs (low numbers of Sox10-negative cells are shown with arrowheads). (d) NCSCs cultured for 14 days, with the final 4 days in 10 ng/ml neuregulin, then immunostained for the Schwann cell marker S100 (occasional S100-negative cells marked with arrowheads). BMSCs were (e) vimentin^+^, (f) CD90^+^, and (g) CD45^-^, consistent with a mesenchymal phenotype. (h) Undifferentiated rat bone marrow cells exhibit a stromal phenotype in culture. (i) Adipogenic differentiation of rat bone marrow-derived stem cells showing the presence of intracellular lipid vacuoles (stained with Oil Red O). (j) Osteogenic differentiation of rat bone marrow showing the presence of calcification (stained with Alizarin Red). Scale bars: 50 μm (b-d), 20 μm (e, f), 10 μm (g), and 200 μm (h-j).

Rat eNCSCs were obtained from the hair bulbs of vibrissa whiskers, as described previously [1,10]. In brief, 2-week-old Sprague-Dawley rat pups were killed by carbon dioxide inhalation and their whisker pads removed and placed in DMEM. Individual whiskers were isolated and trimmed to leave only the bulge region, and the covering connective tissue was removed. Dissected hair-bulge explants were then allowed to attach to collagen-coated T75 flasks for 4 hours in a minimal volume of 10% FCS-DMEM and were subsequently topped up with 5 ml 10% FCS-DMEM. Medium was changed every third day. After 4 to 5 days in culture, neural crest cells were observed migrating away from the hair-bulge explants (Figure 1). At this stage, the hair-bulge explants were carefully removed with an 18G needle to minimize contamination with later-migrating cell types, such as keratinocytes. After 10 days *in vitro*, cells were prelabeled with IO-TAT-FITC nanoparticles and implanted, as described later. Immunocytochemistry at the time of injection demonstrated that more than 60% of the cells were Sox10 immunopositive eNCSCs (Figure 1c). Confirmation of their neuroglial lineage was confirmed by supplementing 10-day-old cultures with 10 ng/ml neuregulin for 4 days, which caused eNCSCs to differentiate into S100-positive Schwann cells (Figure 1d).

Bone marrow-derived stem cells were harvested from adult (300 g) Sprague-Dawley rats. Animals were killed, the hindlimbs were shaved, and the femurs and tibias removed aseptically. Then 10% FCS-DMEM was used to flush the marrow shafts by using a 26G needle, and the bone marrow was harvested. After centrifugation (1,500 rpm, 3 minutes), the pellet was resuspended in 10% FCS-DMEM. Cells were then cultured in previously prepared PLL-coated dishes, in DMEM supplemented with 10% FCS and gentamicin (25 μg/ml). Flasks were incubated at 37°C and 7.5% CO_2_. Two days after plating, the dishes were washed 3 times with 10% FCS-DMEM to remove nonadherent hematopoietic cells. Subsequently, 100% culture medium was changed every 2 days to maintain the health of the culture. To confirm the mesenchymal phenotype of the BMSCs, cells were differentiated into osteogenic or adipogenic lineages for 14 days in culture and were fixed with 4% paraformaldehyde for 10 minutes and stained with either 40 m*M *Alizarin Red (Sigma) or fresh Oil Red O (Sigma) according to Zhao *et al. *[11] (Figure 1i, j). The phenotype of the cells was also confirmed by using vimentin, CD90, and CD45 immunocytochemistry (Figure 1e-g).

### Cell labeling with iron oxide nanoparticles

Dextran-coated iron oxide nanoparticles were prepared in house according to the method of Josephson *et al. *[12]. The nanoparticles were conjugated with both FITC, for histologic analysis and TAT peptide as a membrane-transfection agent. Cells were prelabeled with IO-TAT-FITC nanoparticles (mean diameter, 20 nm)*in vitro *overnight. The final IO incubation solution comprised 10 μg Fe/ml/100,000 cells, in accordance with our previous studies [13,14].

### LPS lesioning and cell implantation

Animals were divided into three groups for each cell type: cells implanted onto the corpus callosum of healthy animals (*n *= 3), cells implanted 3 mm medial to LPS injection (*n *= 5), and cells injected intravenously 1 week after LPS injection (*n *= 2).

**Figure 2:**
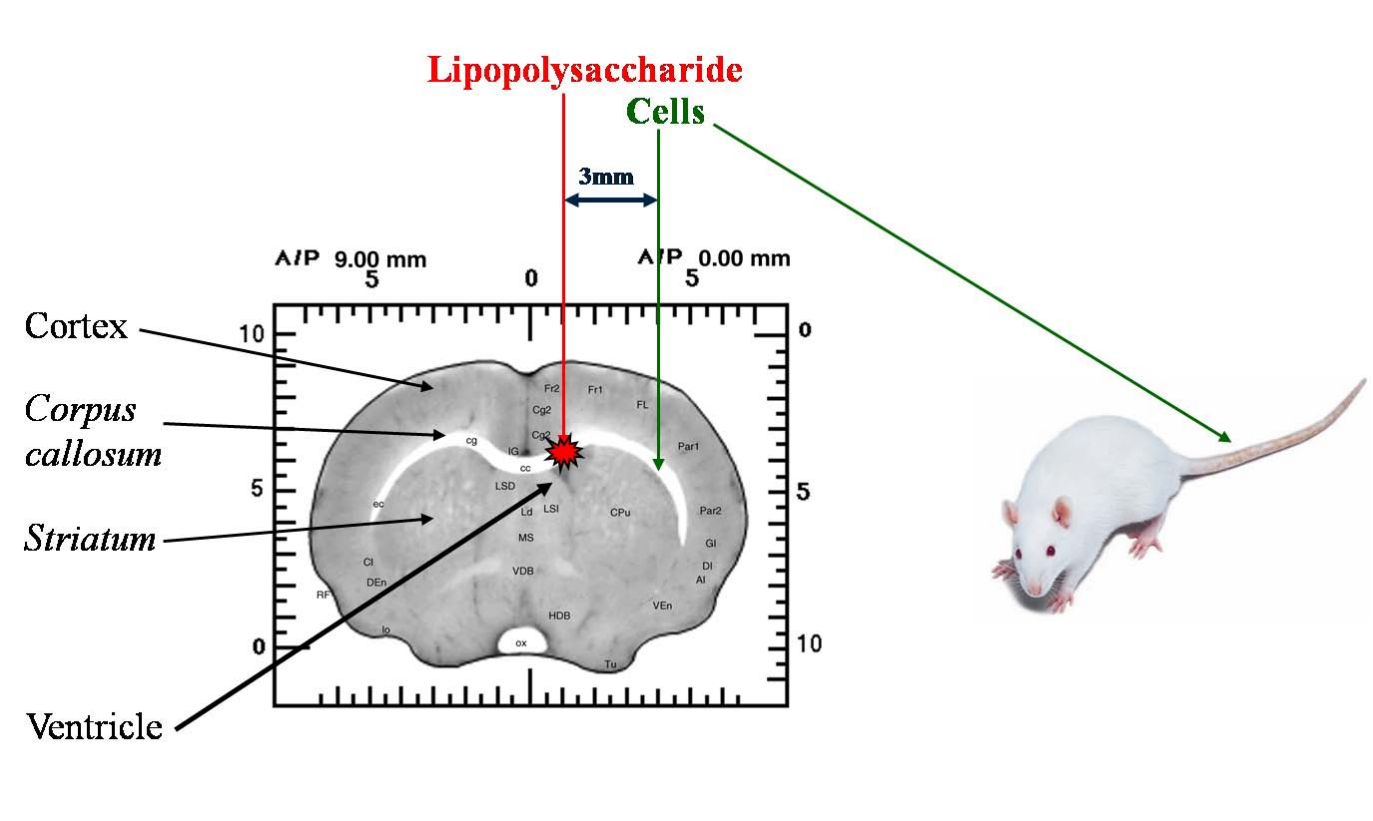
**Localized CNS inflammation model with LPS, with different routes of cell administration**. eNCSCs or BMSCs were either implanted onto the *corpus *callosum of healthy animals, or implanted onto the corpus callosum 3 mm medial to LPS lesion or injected intravenously 1 week after LPS lesioning.

Animals were immunosuppressed with cyclosporine A (Sandimmun, Sandoz, UK) administered intraperitoneally at a dose of 2.5 mg/kg daily from the day of cell implantation until death. Thermoregulation of the rat was monitored during surgery with a rectal probe and kept constant at 37°C with a feedback-controlled warming blanket (Harvard Apparatus, Massachusetts, USA). For the animal lesioning, 5 μl of 1-μg/μl lipopolysaccharide (LPS) (in saline) was injected onto the corpus callosum of healthy animals (250 g) by using a small-animal stereotactic frame (Kopf Instruments, Tujunga, USA) at the following coordinates derived from a rat brain atlas [15]: A-P, + 1.0 mm; L-M, -1.0 mm; D-V, -3.2 mm. Before surgery, a single-cell suspension was prepared in DMEM supplemented with 0.04 mg/ml bovine pancreas DNase (Sigma-Aldrich) to prevent clumping of the cells. Then 50,000 cells in a volume of 5 μl were injected at a rate of 1 μl/min (Figure 2). The needle was maintained *in situ *for 2 minutes before and 5 minutes after cell injection to allow intracranial pressure equalization. For implantation into the healthy animals, stereotactic coordinates were as follows (relative to the bregma); corpus callosum: A-P, + 1.0 mm; L-M, -2.4 mm; D-V, -3.2 mm. When cells were implanted into the LPS-lesioned animals, the following stereotactic coordinates were used: A-P, +1.0 mm; L-M, -4.0 mm; D-V, -4.0 mm to maintain a substantial distance between the cells and LPS.

### Serial MRI

Animals were anesthetised, and thermoregulation was monitored during the MRI. *In vivo *serial imaging was performed at 9.4 T (Oxford Instruments, Abingdon, UK) by using a Varian console (Varian, Palo Alto, CA, USA). The head of the animal was positioned inside a 63-mm (internal diameter) birdcage coil. To visualize the implanted cells, a spin-echo T_2_-weighted image was acquired by using the following parameters: TR = 3,500 msec; TE = 40 msec; matrix = 256 × 128; FOV = 40 × 40 mm; slice thickness = 0.5 mm; with four averages, 20 slices per scan. Each animal was scanned weekly (on average, 4 times in total); with each scan lasting 30 minutes.

T_2 _measurements were obtained by using a spin-echo sequence with a fixed repetition time (TR) and a varying echo time (TE). The following parameters were used: TR = 3,500 msec; matrix = 256 × 128; FOV = 40 × 40 mm; slice thickness = 0.5 mm; number of averages = 1, and varying TEs of 12, 20, 30, 40, 50, 60, 70, 80, and 100 msec. T_2 _maps were reconstructed for each slice by using an ImageJ plug in (11). Regions of interest (ROIs) were drawn on standard anatomic images and then transformed into T_2 _maps. Average signal intensity (SI) values for each ROI at each TE value were transferred to GraphPad Prism (GraphPad Software, La Jolla, CA, USA); T_2 _relaxation curves were then created for each ROI. To visualize and confirm the neuroinflammatory lesion (edema induced by LPS) in the brain, T_2* _fast-spin-echo sequences were used with the following parameters: TR = 3,000 msec; TE_effective _= 24 msec with a train of eight echoes; FOV = 40 × 40 mm; matrix = 256 × 128; averages = 16; slice thickness = 0.5 mm; number of averages = 8.

### Histologic analysis

Animals were intracardially perfused with 4% paraformaldehyde and the brains removed. Brains were cryoprotected in 20% sucrose before being snap-frozen by using precooled isopentane. The 10-μm coronal sections were cut by using a cryostat (Leica Microsystems GmbH, Wetzlar, Germany) and placed on glass slides. The iron oxide nanoparticles were visualized by using a Prussian blue staining procedure by incubating the sections overnight in a solution of 10% potassium ferrocyanide and 20% hydrochloric acid (vol/vol).

### Immunocytochemistry/immunohistochemistry/in situ hybridization

The following primary antibodies were used: rabbit anti-Sox10 (1:10, Calbiochem), rabbit anti-GFAP (1:200, Sigma), CD90 (1:100, BD), CD45 (1:100, BD), S-100β (1:300, Dako), and Vimentin (1:50, Sigma). Cultured eNCSCs and BMSCs were fixed in 4% paraformaldehyde for 20 minutes, permeabilized with 0.1% Triton X-100 in PBS for 10 minutes, blocked with 10% normal sheep serum for 1 hour, and then processed with primary antibody. BMSCs grown in the presence of the osteogenic or adipogenic media were stained with 40 m*M *Alizarin Red or fresh Oil Red O, respectively.

Brain cryosections were blocked in 10% serum (species of secondary antibody), 0.1% Triton-X for 1 hour and then processed with the primary antibody. Primary antibodies (described earlier) were prepared in 10% serum/PBS and incubated overnight at 4°C. The lectin, Isolectin B4, was used to detect microglia (IB4; 1:50, Sigma). The secondary antibody in 10% serum/PBS was added for 1 hour at 4°C. Secondary antibodies were Alexa 488 goat anti-rabbit and Alexa 594 goat anti-mouse (Invitrogen), both applied at a 1:200 dilution. Finally, nuclei were counterstained with DAPI for 10 minutes. Coverslips were mounted on the sections by using Vectashield (Vector Laboratories, Burlingame, USA) as an antifade mountant. Tissue *in situ *hybridization for Sox10 mRNA was performed on the bulge region of vibrissae whiskers, according to standard protocols [16].

## Results

### Phenotypes of donor cells

Neural crest cells characteristically express the nuclear transcription factor Sox10 [1]. Hair-follicle bulge explants were processed for *in situ *hybridization with a Sox10 antisense riboprobe, which revealed a ring of staining corresponding to the niche of adult eNCSCs (Figure 1a). Between 50% and 60% of cultured cells that emigrated from bulge explants within 7 days (Figure 1b) had Sox10-immunopositive nuclei (Figure 1c). None of the cultured cells was immunopositive for the glial cell marker glial fibrillary acidic protein (GFAP; not shown), consistent with previous studies [1]. However, cells could be driven to an S-100 immunopositive glial fate by supplementing 10 day-old cultures with 10 ng/ml neuregulin for 4 days (Figure 1d).

The phenotype of the BMSCs was confirmed with immunocytochemistry and differentiation assays. The harvested BMSCs were found to be vimentin^+^, CD90^+^, and CD45^- ^(Figure 1e-g). The mesenchymal lineage of the rat BMSCs was confirmed by their ability to differentiate readily into adipocytes and osteoblasts [17] (Figure 1h-j).

Previous studies within the group showed that labeling with IO-TAT-FITC does not affect the proliferation or differentiation of BMSCs, nor did it induce apoptosis (data not shown).

### Cell implantation into unlesioned brains

**Figure 3:**
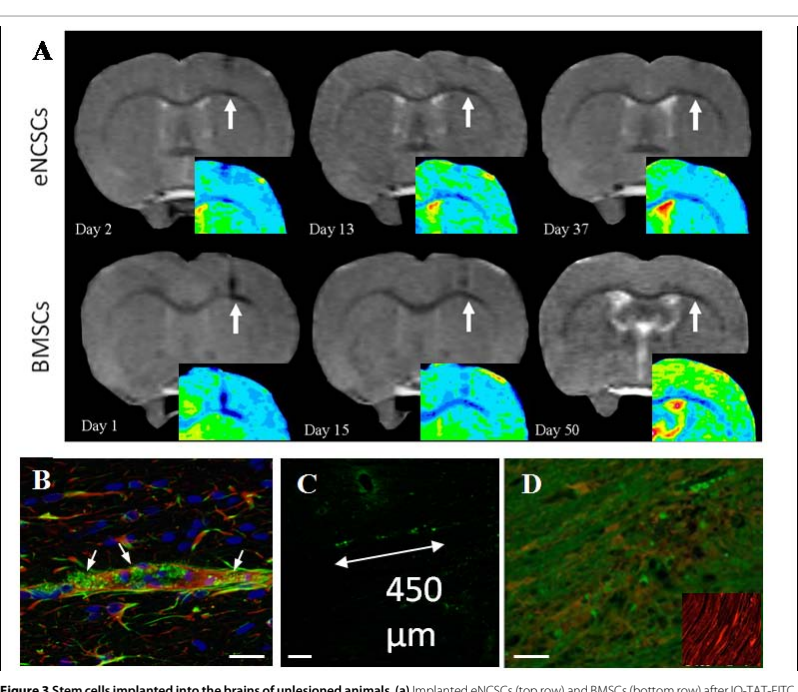
**Stem cells implanted into the brains of unlesioned animals**. **(a) **Implanted eNCSCs (top row) and BMSCs (bottom row) after IO-TAT-FITC labeling and stereotactic implantation (arrows) onto the corpus callosum remained visible by MRI in the same animals for at least 30 days. The spread of the cells appeared to be equally distributed both laterally and medially from the injection site, suggesting no inherent preference of the cells to migrate in one particular direction in the unlesioned brain. False-colored inserts allow better visualization of the hypointense regions. Implanted eNCSCs after IO-TAT-FITC labeling and stereotactic implantation onto the corpus callosum were visible by postmortem histology for a minimum of 30 days. **(b) **Implanted NCSCs (green) on the corpus callosum demonstrated some GFAP (red) glial differentiation (arrows). Nuclear stain DAPI (blue). **(c) **Implanted NCSCs migrate laterally along the white-matter tract of the corpus callosum for approximately 450 μm. **(d) **Implanted NCSCs (green) did not become S-100^+ ^(a marker of mature Schwann cells). Insert shows mature S-100^+ ^Schwann cells in a section of peripheral nerve, immunostained under identical conditions as a positive control. Scale bars: 20 μm (b, d), and 100 μm (c).

Two groups of animals were stereotactically implanted with either eNCSCs or BMSCs (50,000 cells in 5 μl) onto the corpus callosum in the right hemisphere. Both implanted cell types remained visible as an MRI hypointense region for at least 30 days (Figure 3). The cells displayed some lateral and posterior migration away from the injection site along the white-matter tract of the corpus callosum The average lateral spatial distribution measured on MR images for eNCSCs and BMSCs was 0.92 ± 0.36 mm (mean ± SEM) and 1.04 ± 0.11 mm (mean ± SEM), respectively, and this appeared to be spread equally on either side of the injection site. This indicates that the corpus callosum environment does not impose any inherent directionality on the migration of these implanted cells. Previous studies within the group have shown that either implanted labeled dead cells or free iron-oxide nanoparticles are cleared away by the local environment within 30 days, leaving little residual MRI signal void (see Supplementary data 1 in Additional file 1). This suggests that our detection of signal void, of more than 30 days after implantation in the current experiments, most likely represents surviving, live, transplanted cells.

Postmortem histology confirmed the persistence of the implanted FITC^+ ^cells and was anatomically consistent with the MR images (Figure 3a). After direct implantation onto the corpus callosum, the FITC^+ ^eNCSCs were seen to have been distributed longitudinally at a distance of approximately 450 μm (Figure 3c). To determine whether any of the transplanted stem cells had differentiated along the neuroglial lineage, the brain sections were immunostained with glial and neural specific antibodies. Limited GFAP^+ ^differentiation of FITC+ cells (Figure 3b) was noted, but no staining for FITC^+^/βIII-tubulin (not shown); that is, no neuronal differentiation was detected. Moreover, the FITC + implanted eNCSCs showed no evidence of S-100 differentiation (a marker of mature Schwann cells) (Figure 3d). The data are therefore consistent with eNCSC differentiation along a glial pathway, but only to immature nonmyelinating Schwann cells.

### Intracerebral implantation into the LPS-lesion model

**Figure 4:**
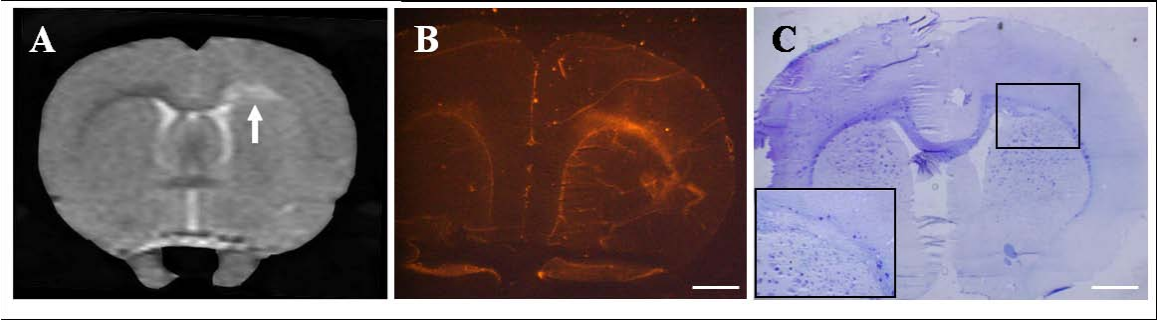
**LPS injection causes an inflammatory focal demyelinated lesion**. **(a) **A T_2*_-weighted MRI sequence was used to visualize the LPS-induced localized inflammatory response, shown as a hyperintense signal (arrow) on the right side of the corpus callosum1 week after lesioning. **(b) **Influx of microglia in the right corpus callosum after injection of LPS, confirmed by isolectin B4 staining (red). **(c) **Demyelinated lesion in the corpus callosum after LPS insult, confirmed by Luxol Blue (blue) and cresyl violet (purple) staining at 1 week. Scale bars: 1 mm (b, c).

**Figure 5:**
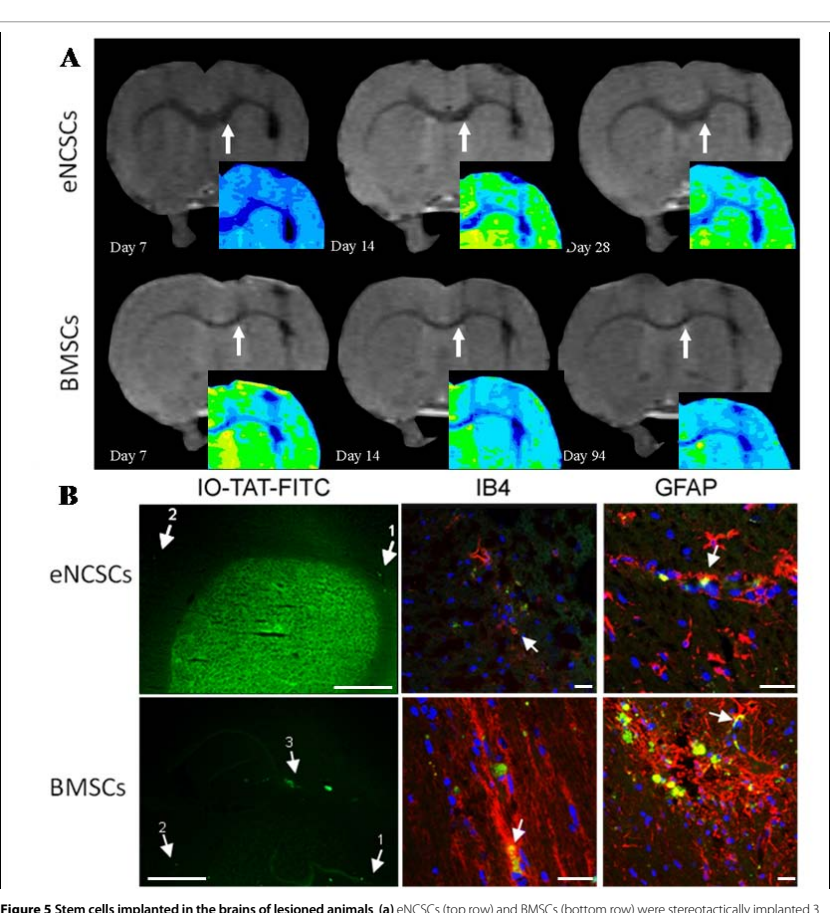
**Stem cells implanted in the brains of lesioned animals**. **(a) **eNCSCs (top row) and BMSCs (bottom row) were stereotactically implanted 3 mm lateral to an LPS inflammatory lesion (arrows). The cells migrated toward the LPS lesion and were seen as a darkening of the right side of the corpus callosum False-colored inserts allow better visualization of the hypointense regions. **(b) **In coronal sections, both IO-TAT-FITC-labeled cell types were observed to have migrated from the implantation site (arrow 1) toward the LPS-lesion site (arrow 2) along the corpus callosum. Some cells were still present along the corpus callosum (arrow 3). IB4 staining (arrow; second column; red) showed that some FITC label had been taken up by microglia. GFAP staining (arrows; third column, red) showed some glial differentiation of implanted cells. Scale bars: 1 mm (b; IO-TAT-FITC), 20 μm (b; IB4, GFAP).

The LPS lesion appeared as a hyperintense signal void on a T_2*- _weighted MR image and led to a demyelination confirmed by absence of Luxol Blue staining (Figure 4). Immediately after injection of LPS, animals were stereotactically implanted with 50,000 IO-TAT-FITC labeled cells (five rats with eNCSCs, three rats with BMSCs). The site of implantation was located 3 mm distally (but a greater distance along the curve of the corpus callosum) from the LPS lesion. *In vivo *MRI imaging at various intervals after implantation demonstrated that both cell types migrated along the white-matter tract of the corpus callosum preferentially toward the LPS lesion, thus demonstrating pathotropic cellular behavior (Figure 5). No evidence was found of cells continuing to migrate beyond the lesion or migrating into the contralateral hemisphere. MR signal void, due to an influx of IO-labeled cells, could be detected in the lesion site for up to 28 days with eNCSCs and for up to 94 days for the BMSCs.

To confirm the differential MR relaxation properties induced by the labeled cells at both the injection and lesion sites compared with the surrounding tissue, a number of varying T_2 _relaxation values were determined. The T_2 _values of different brain regions were determined from the T_2 _maps, generated by varying the echo time (TE) during the MR imaging. It was found that cells at the injection site (26.1 ± 6.3; mean ± SD) and the lesion site (27.3 ± 1.7; mean ± SD) had similar T_2 _values, but these were lower than either the *corpus callosum *(34.0 ± 1.4; mean ± SD) or the *striatum *(45.0 ± 3.8; mean ± SD), thus allowing the cells to be discriminated from the surrounding tissue on MR images thus further confirming migration of the cells toward the lesion from the implantation site (see Supplementary data 2 in Additional file 2).

Postmortem histology of FITC labeling confirmed the MR data that implanted cells had migrated along the corpus callosum to the lesion site (Figure 5). Some eNCSCs were still present at the injection site, and of those cells that had migrated, some exhibited GFAP immunoreactivity, indicating glial differentiation. Around 20% of FITC^+ ^cells were also IB4^+ ^(a microglia/macrophage marker). However, it is unclear whether this represents: differentiation of cells into microglia, engulfment of live cells, dead cells, or of released IO-TAT-FITC nanoparticles. Nevertheless, the remaining population of FITC^+ ^cells were not colocalized with IB4, indicating that the signal void was not from incidental labeling of microglia.

### Intravenous cell injection into an LPS-lesioned model

Having demonstrated that CNS-implanted stem cells migrate to a local inflammatory lesion, it was important to extend these studies to determine whether a less-invasive route for delivery of stem cells toward a central lesion site was feasible. Therefore, 50,000 cells were injected intravenously via the tail vein to assess their migrational potential from the peripheral vasculature to a LPS lesion in the brain.

**Figure 6:**
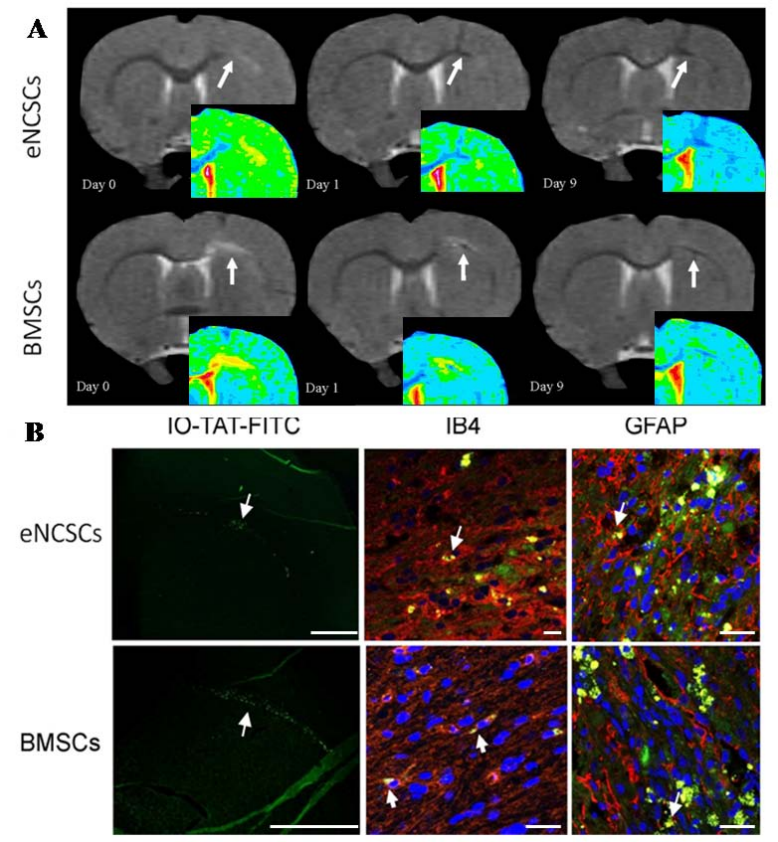
**Stem cells, administered intravenously, home to an inflammatory lesion in the brain**. **(a) **An LPS lesion was induced on the right corpus callosum, followed by intravenous administration of 50,000 IO-TAT-FITC-labelled eNCSCs (top row) or BMSCs (bottom row). Both cell types migrated to the LPS-lesion site (arrows) from the peripheral vasculature within 24 hours of administration. False-colored inserts show better the hypointense regions. **(b) **Both cells types migrated to the LPS lesion after intravenous injection. The IO-TAT-FITC signal demonstrated that the cells had migrated to the lesioned brain area from the peripheral vasculature 24 hours after injection (arrows; first column). Some FITC signal was detected in IB4^+ ^resident macrophages/microglia (arrows; second column; red). Similarly, limited differentiation into GFAP^+ ^glia was seen (arrows; third column; red). Scale bars: 1 mm (b; IO-TAT-FITC), 20 μm (b; IB4, GFAP).

Within 24 hours after intravenous administration of stem cells, a MR signal void developed at the site of the LPS injection/lesion (Figure 6). This signal void became more defined over the following 8-day period (Figure 6).

Animals were killed 10 days after administration of the IO-TAT-FITC-labeled cells. In one animal, the number of IO-TAT-FITC^+ ^cells, on alternate tissue sections (to reduce the possibility of double counting), was counted at the LPS-lesion site. It was estimated that approximately 4,000 of 50,000 cells administered intravenously had homed to the LPS-lesioned area from the peripheral vasculature, thus representing 8% of the total eNCSCs injected. Furthermore, the cells were found to have spread along the corpus callosum (Figure 6).

The FITC^+ ^BMSCs also were found to be distributed throughout the right corpus callosum over a distance of 3.6 mm around the LPS-demyelinated lesion area. An issue with cell-implantation studies is whether the MRI reports on either nanoparticle-labeled cells or free IO-TAT-FITC nanoparticles released on cell death and subsequently engulfed by resident microglia or transient macrophages, which then migrated to the lesion. Isolectin-B4 staining indicated that a small proportion (about 20%) of the cells were double positive for both FITC and IB4. However, most FITC^+ ^cells were IB4^-^, suggesting that the majority of cells within the lesion site were the peripherally transplanted stem cells that had migrated to the lesion from the circulation.

## Discussion

Increasing clinical interest exists in the use of transplantable stem cells as a means of repairing neurodegenerative disease or for gene delivery. The success of such approaches will be dependent on not only the source of donor stem cells and their expansion *in vitro*, but also their mode of delivery, where localized surgical delivery to the brain carries inherent risks of morbidity. Studies have begun to evaluate the clinical efficacy of using intravenously administered bone marrow stem cells in diseases such as spinal cord injury [18]. This study demonstrates that MRI is a useful technique that allows longitudinal noninvasive monitoring of stem cell migration in the brains of individual animals, giving important information on cell dynamics and interactions with the host CNS.

Implanted eNCSCs or BMSCs were visible as a hypointense region in T_2 _MRI images, allowing them to be tracked as they migrated within the brain. Implanted cells remained visible at the lesion site by MRI for the duration of the experiment (up to 13 weeks). Stem cells implanted directly into the brain were found to migrate preferentially along the major white matter tracts in both healthy and lesioned animals. In animals with a focal inflammatory lesion, the transplanted cells displayed unidirectional pathotropic migration to the lesion, where they ceased migrating and remained visible on MRI for several months. Moreover, and of potential benefit for clinical applications, both cell types similarly displayed migration to a focal inflammatory brain lesion from the peripheral vasculature.

Several studies have demonstrated the migration of stem cells after intravenous administration in models of neurodegeneration [19-22]. However, those models involved cerebral ischemia and compression injury, which result in a more severe lesion. Our study has shown that relatively minor, focal lesions provide sufficient cues to attract stem cells from the peripheral vasculature. Our LPS-lesion model induces a focal demyelination, resulting in an influx of microglia; such lesions have been shown to stimulate the release of cytokines such as IL-1β, TNF, and IL-6 [23], which may have provided the chemoattractant signals for eNCSCs and BMSCs. The migration of transplanted stem cells through the brain parenchyma may also be aided by their secretion of matrix metalloproteases (MMPs), because BMSCs require MMP1 to migrate toward human gliomas [24]. Because no donor cells could be detected in other regions of the brain, this suggests that the cells were not simply trapped within the cerebral blood vessels, but were responding specifically to chemotactic factors released from the lesion. It has been reported that eNCSCs are not migratory when transplanted into the lesioned spinal cord [25], whereas our work shows that eNCSCs have homing properties similar to those of BMSCs and invade brain lesion from the peripheral vasculature. This suggests that the local CNS environment may have an important effect in regulating NCSC attraction and invasion, possibly by altering the expression of NCSC chemokine receptors or proteases essential for cell migration.

Although the intravenous administration route has obvious clinical advantages, we found that one challenge of using this route will be to get sufficient cells to the lesion for therapeutic benefit. Thus, we estimate that only about 8% of the intravenously injected stem cells migrated to the LPS lesion. By contrast, more than 10 times this number of stem cells reached the lesion site after intracranial administration some 3 mm from the lesion site. It will be of interest to determine whether other vascular routes, such as the carotid artery, might increase the proportion of injected stem cells that enter the brain-lesion site. It will also be of interest to examine whether stem-cell engraftment can be maximized by changing the postlesion timing of cell delivery. Any delay in delivery, sufficient to permit the *in vitro *expansion of autologous stem cells, would obviously be desirable from a practical and clinical perspective.

Concerning the fate of the administered stem cells, we found that BMSCs and eNCSCs were able to adopt glial fates in the brain, although we did not detect stem cell-derived neurons. A previous study demonstrated that BMSCs have the ability to differentiate into astrocyte-like and oligodendrocyte-like cells after implantation into an ischemic lesion [17]. A separate study, by using eNCSCs in a in rat spinal cord crush lesion model, detected limited S-100 differentiation of implanted eNCSCs, indicative of mature Schwann cells [25]. We did not detect S-100 differentiation of the implanted eNCSCs in our focal LPS-lesion model, although several implanted cells expressed GFAP, an early marker of glial differentiation for both astrocytes and immature Schwann cells [26]. It remains unclear whether, had the experiment been allowed to proceed for a longer period, the implanted eNCSCs in our model would eventually mature into myelinating Schwann cells.

Previous studies within the group have shown that dead cells, prelabeled with IO-TAT-FITC, are cleared away from the local area and do not contribute to a signal void (see Supplementary data 1 in Additional file 1). In addition, and consistent with this study, previous studies using live stem cells have shown that whereas a few of the cells are engulfed by IB4^+ ^microglia, the majority remain nonphagocytosed after BMSC implantations into a rodent model of Parkinson disease [14].

## Conclusions

In summary, this study has shown by using a noninvasive longitudinal *in vivo *imaging modality, that two stem-cell types, with the potential for autologous cell therapy and gene targeting in CNS injury and neurodegenerative diseases, have pathotropic properties in a focal demyelinating brain lesion when they are administered by either the intracerebral or the intravenous routes. Moreover, this work reinforces that intravenous delivery is a viable clinical option for the delivery of cell-based therapies, with obvious clinical advantages over direct CNS injection.

## Abbreviations

BMSC: bone marrow stem cell; DMEM: Dulbecco modified Eagle medium; DRG: dorsal root ganglion; eNCCs: epidermal neural crest cells; FCS: fetal calf serum; GFAP: glial-fibrillary acidic protein; HSC: hematopoietic stem cell; IB4: isolectin B4; IO-TAT-FITC: iron oxide transactivator of transcription fluorescein isothiocyanate; LPS: lipopolysaccharide; MRI: magnetic resonance imaging; MSC: mesenchymal stem cell; NCC: neural crest cell; TE: echo time.

## Competing interests

The authors declare that they have no competing interests.

## Authors' contributions

JJ, JG, and KB designed the study. JG and JJ carried out the *in vitro *cell work. JJ carried out the *in vivo *work. CC designed and developed the MR imaging. WJ designed and synthesized the iron oxide nanoparticles. All authors contributed to and approved the manuscript.

## Supplementary Material

Additional file 1 **Signal void after cell implantation**. Live (arrows) and dead (arrowheads) BMSCs were injected into the right and left striata, respectively. The signal void from the live cells remained for 53 days with little remaining signal void from the implanted dead cells. Click here for file

Additional file 2 **Signal-intensity decay and map of T_2 _values in the rat brain**. (a) Signal-intensity decay of different regions of interest (ROIs) at different TEs, showing the different MR-relaxivity properties of different brain regions at 14 days after the lesion and after cell implantation. Map of T_2 _values (ms) within the rat brain. **(b) **Because of the superparamagnetic properties of iron oxide particles, labeled cells induced a decrease of T_2 _value at the site of implantation (arrow 1), and at the LPS lesion site (arrow 2). Click here for file
