# Supplementary figures and images for: Homing of stem cells to sites of inflammatory brain injury after intracerebral and intravenous administration: a longitudinal imaging study
Source: Stem Cell Res Ther. 2010 Jun 15;1(2):17. doi: 10.1186/scrt17 (PMC2905093; doi:10.1186/scrt17)

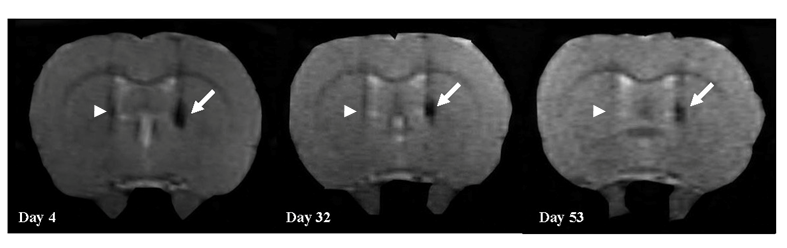

Supplement: Additional file 1 — Signal void after cell implantation. Live (arrows) and dead (arrowheads) BMSCs were injected into the right and left striata, respectively. The signal void from the live cells remained for 53 days with little remaining signal void from the implanted dead cells. [file scrt17-S1.TIFF]

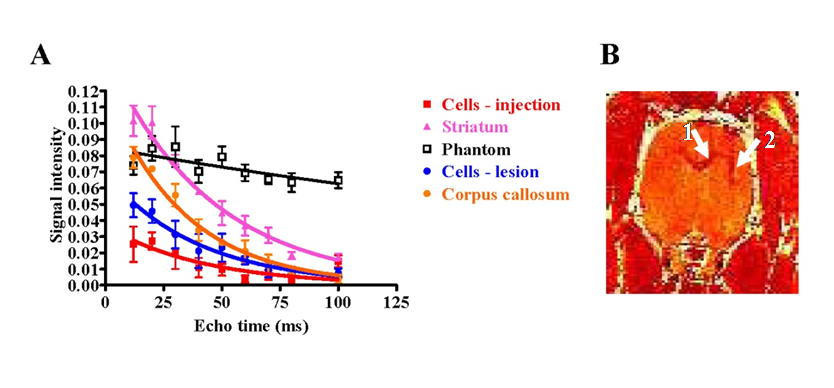

Supplement: Additional file 2 — Signal-intensity decay and map of T2 values in the rat brain. (a) Signal-intensity decay of different regions of interest (ROIs) at different TEs, showing the different MR-relaxivity properties of different brain regions at 14 days after the lesion and after cell implantation. Map of T2 values (ms) within the rat brain. (b) Because of the superparamagnetic properties of iron oxide particles, labeled cells induced a decrease of T2 value at the site of implantation (arrow 1), and at the LPS lesion site (arrow 2). [file scrt17-S2.TIFF]
